# Severe Ovarian Hyperstimulation Syndrome in a Woman With Breast Cancer Under Letrozole Triggered With GnRH Agonist: A Case Report and Review of the Literature

**DOI:** 10.3389/frph.2021.704153

**Published:** 2021-07-06

**Authors:** Giuseppe Gabriele Iorio, Marika Ylenia Rovetto, Alessandro Conforti, Luigi Carbone, Roberta Vallone, Federica Cariati, Francesca Bagnulo, Raffaella Di Girolamo, Antonio La Marca, Carlo Alviggi

**Affiliations:** ^1^Department of Neuroscience, Reproductive Science and Odontostomatology, School of Medicine and Surgery, University of Naples Federico II, Naples, Italy; ^2^Department of Obstetrics and Gynecology, Center for Fetal Care and High-Risk Pregnancy, University of Chieti, Chieti, Italy; ^3^Department of Medical and Surgical Sciences for Children and Adults, University of Modena and Reggio Emilia, Modena, Italy

**Keywords:** ovulation induction, OHSS, breast cancer, fertility preservation, GnRH agonist, vascular endothelial growth factor

## Abstract

We report a rare case of ovarian hyperstimulation syndrome (OHSS) in a 28-year-old woman with breast cancer and with a history of polycystic ovary syndrome (PCOS) despite treatment with letrozole and gonadotropin-releasing hormone agonist (GnRH-a) triggering in a GnRH antagonist (GnRH-ant) protocol without the administration of any human chorionic gonadotropin (hCG) for luteal-phase support. The patient, who underwent controlled ovarian syndrome (COS)-oocyte cryopreservation before chemotherapy, required hospitalization. Complete recovery was achieved after treatment with volume expanders, human albumin, and cabergoline. Based on our case and literature review, it is possible to establish that estradiol (E_2_) modulation with letrozole and GnRH-a triggering does not eliminate the risk of OHSS. Furthermore, it is advisable to postpone GnRH-a *depot* to minimize the risk of OHSS after the suspension of letrozole, following menstruation or at least 7–8 days after triggering. It would be desirable to identify high-risk patients, also on a genetic basis, in order to avoid delays in oncologic treatments that could strongly impact life expectancy.

## Introduction

Ovarian hyperstimulation syndrome (OHSS) is a severe complication associated with controlled ovarian stimulation (COS) during assisted reproductive technology (ART) and occurs in approximately 1–5% of treatment cycles (1). OHSS is characterized by ovarian enlargement, ascites, hemoconcentration, hypercoagulability, and electrolyte imbalances. It appears with abdominal distension and discomfort, nausea, vomiting, dyspnea, and diarrhea. Most severe clinical presentations may lead to acute renal insufficiency, pleural effusion, and venous thromboembolism (1). Although human chorionic gonadotropin (hCG), estradiol (E_2_), and vascular endothelial growth factor (VEGF) represent the main actors of OHSS, the former seems to be the trigger of the process (2, 3). There are several associated risk factors such as young age, lower body mass index (BMI), polycystic ovary syndrome (PCOS), and elevated anti-müllerian hormone (AMH) values (4).

Ovarian hyperstimulation syndrome is considered an hCG-dependent phenomenon. According to the classical concept, neither early nor late OHSS can occur if exogenous hCG is not administered. Although it is widely accepted that triggering with gonadotropin-releasing hormone agonist (GnRH-a) represents the most efficient prevention in the high-risk patient (5, 6), several cases of early and severe OHSS following this approach are described in the literature study (7–14). The role of estrogens in the pathogenesis of OHSS is still a matter of debate. Despite not being the direct cause of the syndrome, a predicting role of E_2_ levels during COS has been documented (15, 16). More specifically, E_2_ values in patients with OHSS were >3,500 pg/ml (1).

Premature ovarian insufficiency (POI) is common in young women with cancer due to the therapies. Chemotherapy is the leading cause of follicular damage, which depends on age, ovarian reserve, chemotherapeutic drugs, and doses. The growing interest in treatments aimed at fertility preservation in patients with cancer has led to new protocols. In order to limit high hormone concentrations, letrozole has been approved to reduce estrogen levels in women affected by hormone-sensitive cancers (17). Furthermore, the use of GnRH-a to trigger oocyte maturation prior to egg retrieval during GnRH antagonist (GnRH-ant) protocol is recommended (1). These stimulation regimens produce results comparable to standard protocols associated with significantly lower E_2_ levels (18). We report a case of OHSS that occurred in a woman with breast cancer following COS performed in co-treatment with letrozole and triggered with GnRH-a. Furthermore, a review of similar cases reported in the literature studies was performed. All procedures were conducted in conformity with the Declaration of Helsinki, and the patient signed an informed consent form.

## Case Report

A 28-year-old woman, diagnosed with breast cancer, did a consultation for fertility preservation at our oncofertility clinic (University of Naples Federico II). The patient had a history of oligomenorrhea and PCOS. Clinical features of the patient are reported in Table 1. Transvaginal ultrasonography (USG-TV) revealed polycystic ovaries and central hyperthecosis (19). She had no family history of breast and/or female genital tract tumors. In order to preserve her oocytes, the patient underwent COS and oocyte cryopreservation. The procedure was performed between breast surgery and chemotherapy. A unilateral quadrantectomy was performed, and subsequently, the patient began treatment with letrozole (Femara, Novartis Pharma S.p.A., Varese, Italy; 5 mg daily). The ovarian stimulation protocol is shown in Table 2. On the 11th day after stimulation, about 20 follicles were aspirated. A total of 16 oocytes were retrieved, and 13 mature oocytes were cryopreserved. At the post-pick up (PU) ultrasound check, there was a flap in the Douglas (26 mm x 16 mm) and there was an anterior-free fluid collection (34 × 39 mm). Her hemoglobin (Hb), hematocrit (Ht), and white blood cell count (WBC) values were 12.8 g/dl, 38.1%, and 5,200/μl, respectively. Treatment with letrozole was suspended on the day of GnRH-a triggering and was restarted after collecting eggs until E_2_ levels were below 50 pg/ml. On the day after PU, triptorelin (Decapeptyl, Ipsen S.p.A., Milan, Italy; 3.75 mg/28 days) was administered for ovarian protection during the chemotherapy, which the patient would have started on the following day. E_2_ and progesterone levels on the 5th day after the administration of triptorelin *depot* (17th after starting COS) were 195 pg/ml and 562 ng/ml, respectively.

**Table 1:** Patient's clinical features.

Age	28 years
Ethnicity	Caucasian
BMI	21.1 kg/m^2^
Menarche	13 years
Breast cancer type	NST triple negative (PR-/ER-/Her2Neu -)
Grading (Elston-Ellis)	G3

**Table 2:** Ovarian stimulation protocol.

**COS**	**Drug**	**Dose**	**E_**2**_**	**Progesterone**	**Follicles**	
**(day)**			**(pg/ml)**	**(ng/ml)**	**R ovary**	**L ovary**
−2	Letrozole (Femara, Novartis Pharma S.p.A. Varese, Italy)	5 mg/day				
0(12th cycle day)	rFSH (Gonal F, Merck Serono Ltd, UK)	150 IU/day (the dose was not modified during COS)			Polycystic with a dominant follicle of 10 mm	Polycystic
4	Ganirelix (Fyremadel, Ferring S.p.A., Milano, Italy)	0.25 mg/day			1 (19 mm) 6 (11–13 mm) 2 (<10 mm)	3 (10–12 mm) 5 (<10 mm)
6			628	0.7	1 (24 mm) 1 (17 mm) 5 (10–13 mm)	2 (15 mm), 6 (11–13 mm) 1 (<10 mm)
9	Triptorelin acetate (Fertipeptil, Ferring S.p.A., Milano, Italy)	0.2 mg	1,747	3.14	10 (16–20 mm)	10 (16–20 mm)

On the 18th day, the patient came to the hospital emergency room complaining of abdominal bloating, pelvic pain, and mild dyspnea. On physical examination, ascites were found. Hospitalization was required. Her Hb, Ht, and WBC levels were 15.7 g/dl, 47.3%, and 13,240/μl, respectively. USG-TV revealed markedly enlarged ovaries (70 mm × 55 mm, bilaterally) and significant anterior (70 × 50 mm) and posterior (70 mm x 60 mm) free fluid. Furthermore, the collection of a subphrenic (82 mm) and subhepatic (119 mm) fluid was found.

The patient was treated with volume expanders (tetramido 500 ml/day), prophylactic anticoagulation (enoxaparin sodium 4,000 UI/day), and antibiotic therapy (cefazoline 2 g/day). The following day, 40 g of human albumin was administered. Subsequently, once Ht was <37%, 20 mg of furosemide and 0.5 mg cabergoline were added to therapy. After 5 days, probably due to chemotherapy, the patient developed severe leukopenia (530/μl), for which filgrastrim (1/day for 7 days) was started. Fluid therapy and cabergoline were continued for 12 days. She was discharged on the 30th day after starting COS when Hb, Ht, and WBC levels were 8 g/dl, 23.9%, and 17,420/μl, respectively. On ultrasound, the size of the ovaries and the free fluid were reduced, with the disappearance of the collection of subhepatic and subphrenic fluid. Her chemotherapy regimen was scheduled for the same day.

## Discussion

We report a case of OHSS arising in a patient who received COS in association with letrozole and GnRH-a triggering. More specifically, severe OHSS occurred in the high-risk patient (young age, low BMI, and PCOS), despite modulated E_2_ peak (1,747 pg/ml), and all the precautions were put in place to prevent the syndrome. In fact, letrozole is considered a valid option for modulating E_2_ levels, whereas GnRH-ant protocol associated with GnRH-a triggering represents a safer way of performing COS in both patients with PCOS and those with breast cancer (20–22). The day after ovum PU, the patient restarted letrozole administration and received triptorelin *depot* 3.75 mg. The OHSS occurred on the 6th day after the *depot*.

Several cases of different grade of OHSS have been reported following treatment with GnRH-ant associated with GnRH-a triggering in non-oncologic patients (7–14). These cases raise questions about the dogma that the presence of exogenous or endogenous hCG is required for the onset of OHSS. Possible explanations for these observations include variations of genes, such as allelic variants of hCGs and their receptors, estrogen receptors, and VEGF gene and its receptor.

Some differences characterized our case: The syndrome occurred in a woman with breast cancer who received co-treatment with letrozole for modulating estrogen levels. An analogous observation was reported by Kim *et al*. The authors described a patient (35 years old, BMI 19.3 Kg/m^2^, AMH 12 ng/ml), with a history of PCOS, who developed severe OHSS following stimulation conducted with recombinant follicle-stimulating hormone (rFSH) (Gonal F, Serono Inc., Rockland, MA; 225 UI daily) and letrozole (Femara, Novartis Pharma S.p.A., Italy; 5 mg) despite low E_2_ levels (636 pg/ml) (23). The main difference between the two cases was that the triggering was performed with GnRH-a in our case and hCG (Ovidrel, Serono Inc., Rockland, MA; 250 μg) in the other case. The appearance of OHSS in patients receiving letrozole seems to reinforce the idea that modulating E_2_ levels, despite necessary for preventing cancer progression, have an unpredictable impact on the prevention of OHSS. Papanikolaou et al. had already shown that E_2_ levels are less reliable in predicting OHSS (24). Sen et al. have shown that androgens not only increase the expression of FSH receptor (FSHR) on the follicles and, consequently, increase the growth and development of the follicle mediated by FSH but also prevent follicular atresia by inducing the expression of an antiapoptotic microRNA (miR-125b). Furthermore, these authors observed a positive effect of androgens on ovulation rates in women with reduced ovarian reserve. This finding could explain the unregulated follicle growth observed in a disease with excess androgen, such as PCOS (25). The dosage of androgen levels in the days preceding the administration of GnRH-a *depot* could be useful in the decision-making process.

Another important observation is related to the administration of GnRH-a *depot* after ovum PU and during the “luteal phase” administration of letrozole. The finding that letrozole addition in the luteal phase decreases serum estrogen levels of patients after oocyte retrieval but cannot delete the risk of severe OHSS that had been previously reported (26). Recently, three cases have been reported in which, despite GnRH-a triggering and use of letrozole, the administration of GnRH-a *depot* shortly after PU led to OHSS (Table 3) (27, 28). As in our case, the onset of OHSS followed the use of GnRH-a *depot*, administered for chemoprotection, after PU.

**Table 3:** Cases of OHSS reported after GnRH agonist depot.

**Case**	**Age**	**BMI**	**AMH**	**AFC**	**Risk factors**	**Cancer type**	**Menstrual phase at the beginning of the protocol**	**Stimulation protocol**	**Triggering**	**E_**2**_ on triggering day**	**Long-acting GnRH agonist**	**OHSS onset**
	**(years)**	**(Kg/m^**2**^)**	**(ng/ml)**							**(pg/ml)**		
(27)	28	22.5	8.06	39	PCOS	Breast cancer	Follicular	150 IU/day rFSH; 0.25 mg/day ganirelix; 5 mg/day letrozole	0,2 mg triptorelin	518	3.75 mg depot triptorelin 2 day after PU	3rd after depot
(28)	39	38	11.6	32	PCOS	Breast cancer	Random start	A total of 3,525 IU of gonadotropin; 0.25 mg/day cetrorelix; 7.5 mg/day letrozole	4 mg leuprolide	581	11.25 mg depot leuprolide the day after PU	4th after depot
	33	NA	8.1	43	PCOS	Colorectal cancer	Random start	A total of 2,275 IU of gonadotropin; 0.25 mg/day cetrorelix; 7.5 mg/day letrozole increased to 10 mg/day for the last 2 days	4 mg leuprolide	949	3.75 mg depot leuprolide the day after PU	6^th^ after depot

*AMH anti-mullerian hormone; NA not available; PCOS polycystic ovary syndrome; PU ovum pick-up*.

The waiting duration before safely administering GnRH-a *depot* after PU is not well established, but the authors assumed that waiting 7–8 days before the administration may be sufficient to avoid an OHSS.

However, it is interesting to note that, in the case reported by Friedler *et al*., OHSS occurred after the administration of a GnRH-a, which is used as luteal phase support (LPS) (8).

Regarding the LPS, it is important to introduce the concept of “luteal costing,” which could be adapted to patients with cancer (29, 30). The idea of individualizing the LPS by monitoring serum progesterone levels could be used in these patients to identify the best timing for the administration of GnRH-a *depot* to prevent the onset of OHSS.

Although an etiology linked to hCG has been ruled out, the molecular mechanisms that triggered the syndrome remain unclarified. A pathogenic role for the VEGF system has been also hypothesized. Stouffs *et al*. analyzed the DNA samples of three women from the studies of Gurbuz *et al*. and Santos-Ribeiro *et al*. that have been reported with a rare variant in VEGFR3 (9, 13, 31). The study of genetic variants of VEGF receptors could open new perspectives on the mechanisms involved in the syndrome. Borgwardt et al. (32) evaluated the presence of genetic variants predisposing to the development of OHSS and found an association with the integrin-linked kinase (ILK) signaling pathway (2020). ILK signaling stimulated VEGF expression in tumor cells and VEGF-mediated endothelial activation (33). ILK was identified as a critical regulator of VEGFR3 signaling (34).

According to the literature review, PCOS is the most common risk factor. PCOS is characterized by ovarian abnormal vascularization and significant differences on levels of multiple pro-angiogenic factors (VEGF, placental growth factor, angiopoietins, transforming growth factor-beta, platelet-derived growth factor, and basic fibroblast growth factor) (35). In a normal ovary, vascular homeostasis is highly regulated by Notch signaling, which inhibits ovary angiogenesis and maintains the integrity of the ovarian vasculature (36). Notch activation induces increased expression of VEGFR1 and soluble VEGFR1 in cultured endothelial cells and provides negative feedback on the activity of the VEGF/VEGFR2 pathway (37). Furthermore, the Notch pathway seems to affect the expression of VEGFR3 through regulating VEGFR3 promoters. Therefore, it is feasible to hypothesize that the Notch pathway is dysregulated in PCOS. Future research studies are needed to establish a clinically useful connection between the receptor mutations and the occurrence and intensity of OHSS.

It is also not possible to exclude that, in patients with cancer, there is a higher risk profile linked to increased vascular permeability and angiogenic stimulus.

Youssef et al. reported that cabergoline, a dopaminergic agent that blocks the binding of VEGF to the VEGF receptor, prevented an increase in vascular permeability and reduced the incidence and severity of the syndrome, without completely preventing it when hCG was administered (38). The best time to start administration of dopaminergic agents is a few hours before triggering (11). Administration of cabergoline as a prophylactic measure against OHSS in high-risk patients appears to be effective without compromising pregnancy rates (39).

In conclusion, at the moment, we can hypothesize that the onset of OHSS in this case was related to the administration of GnRH-a *depot* shortly after ovum PU. However, on the basis of this case and the other three previously reported cases, it is not yet possible to identify the molecular mechanism underlying the syndrome. In addition, we were unable to identify parameters that suggest the onset of the syndrome. Is it possible that the GnRH-a *depot* acts on some peripheral receptors? (40).

Anyway, as a precaution, it is possible to suggest that [1) OHSS is still possible even after modulating estrogen production with letrozole and using GnRH-ant and that the use of GnRH-a triggering is to be preferred due to the necessity to prevent comorbidities and delays in adjuvant therapy and [2) it is advisable to postpone GnRH-a *depot* for minimizing the risk for OHSS. Should it be the case, an ideal timing has to be identified (i.e., after the suspension of letrozole, following menstruation, or at least 7–8 days after triggering). Finally, it would be desirable to identify high-risk patients, also on a genetic basis, in order to avoid delays in oncologic treatments that could strongly impact life expectancy.

## Data Availability Statement

The raw data supporting the conclusions of this article will be made available by the authors, without undue reservation.

## Ethics Statement

Written informed consent was obtained from the individual(s) for the publication of any potentially identifiable images or data included in this article.

## Author Contributions

GI and CA contributed to the conception and design of the study. MR, RV, RD, FC, and FB collected the clinical data. GI and MR reviewed the literature. GI and MR wrote the first draft of the manuscript. LC and AC wrote sections of the manuscript. GI, AL, and CA did the final editing of the manuscript. All authors contributed to the review of the manuscript, read and approved the version sent.

## Conflict of Interest

The authors declare that the research was conducted in the absence of any commercial or financial relationships that could be construed as a potential conflict of interest. The reviewer HF declared a past co-authorship with one of the authors AL to the handling Editor.
